# Glyceryl trinitrate has opposite effects on different experimental models of pain

**DOI:** 10.1186/1129-2377-14-S1-P90

**Published:** 2013-02-21

**Authors:** VA Grigoraay, A Luca, A DondaÅŸ, T Alexa, OC Mungiu, CR Bohotin

**Affiliations:** 1 Department of Neurology, Recovery Hospital, Grigore T Popa University of Medicine and Pharmacy Iasi, Romania, Romania; 2 Centre for the Study and Therapy of Pain, University of Medicine and Pharmacy, Grigore T Popa University, Iasi, Romania, Romania

## Introduction

The actions of glyceryl trinitrate (GTN) are the result of its bioconversion into NO; NO increases the intracellular concentration of cyclic guanosine monophosphate (cGMP), which produces pain modulation in the central and peripheral nervous system ( Griesberger et al. 2011 ). In addition, GTN administration is considered a reliable experimental model of migraine, based on the neuronal effects on the integrative-nociceptive structures ( de Tommaso et al. 2004 ).

## Purpose

to investigate the effects of GTN 4 hours after its administration on different experimental pain models in mice.

## Methods

Sixteen Swiss male mice were divided into 2 groups: control group (n=8) and GTN group (n=8, 10 mg/kg b.w. i.p ). Assessment of locomotor activity (activity cage) and nociceptive tests (tail flick-TF and hot plate-HP) were performed before GTN administration and considered as baseline. Four hours after GTN injection, locomotor activity assessment and nociceptive tests were re-evaluated; afterwards, 20 Î¼l of 5% formalin were administrated into the upper right lip in order to assess formalin-induced orofacial pain. The results were compared with paired and unpaired Student’s t test.

## Results

GTN administration significantly increased HP latencies (p=0.0002) and showed a tendency towards increasing TF (p=0.056). A decrease in the locomotor activity was noted for both vertical movement activity (-78% p =0.001) as well as horizontal movement activity (-87% p=0.0001). GTN had no significant effect in influencing formalin-induced orofacial pain response.

## Conclusion

In our study GTN administration in mice exerted analgesic effects on acute nociception but had no effect on orofacial formalin pain. In addition, GTN decreased locomotor activity. Taken together, our results demonstrate that trigeminal pain is differently modulated by GTN as compared to nociception in TF and HP.
